# Supplementary material for: Human basal-like breast cancer is represented by one of the two mammary tumor subtypes in dogs
Source: Breast Cancer Res. 2023 Oct 3;25:114. doi: 10.1186/s13058-023-01705-5 (PMC10546663; doi:10.1186/s13058-023-01705-5)
Supplement: Supplementary file 1 — Additional file 1 Fig. S1. RNA-seq quality control. Fig. S2. Validation of canine mammary tumor subtyping results shown in Fig. 1 using different strategies and data set. Fig. S3. Dog-alone and cross-species PAM50 classification using canFam4 and its annotation; hBLBC- and hLumA feature gene identification via machine learning. Fig. S4. Differentially expressed (DE) gene analysis indicates the enrichment of hBLBC signatures in cBLMT of the validation set. Fig. S5. Canine tumors, especially cBLMTs, express PGR more abundantly than hBLBCs. Fig. S6. Purine de novo synthesis and serine synthesis are more activated in cBLMTs and hBLBCs, compared to normal mammary tissues. Fig. S7. PGR, but not ESR1 or PRLR, correlates with several T-cell exhaustion signature genes in mRNA expression in ER-PR+ cBLMTs; PRL and PRLR expression in canine and human tumors. Fig. S8. PRLR positively correlates with many genes in mRNA expression, but is not associated with gene silencing in cBLMT [file 13058_2023_1705_MOESM1_ESM.pdf]

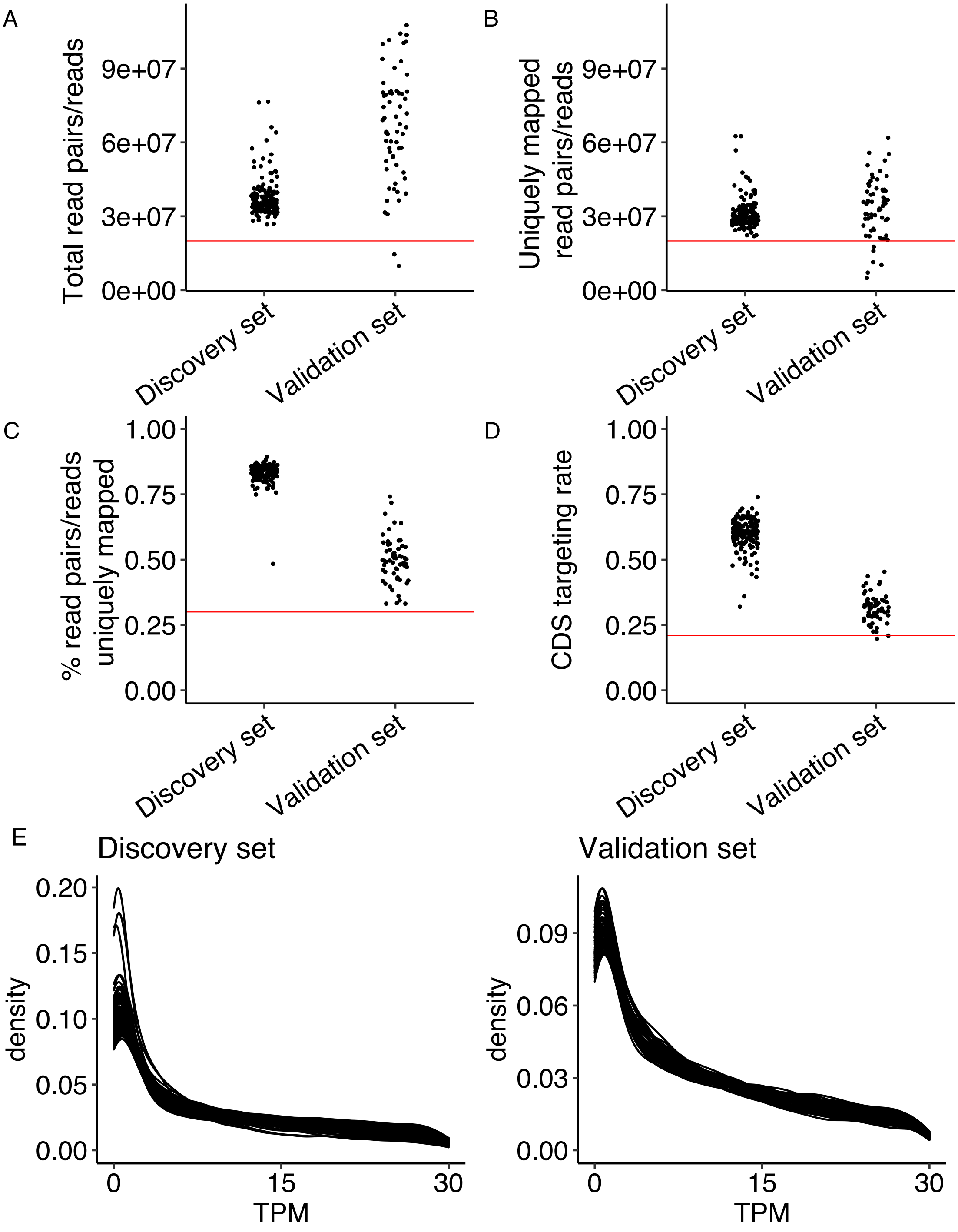

Fig. S1. RNA-seq quality control; related to Fig. 1 and Table S1.

A. Distributions of sequencing amounts of the discovery set (the number of read pairs per sample) and the validation set (the number of reads per sample). Each dot

represents a sample. Red line specifies the cutoff (20 million total reads) and samples below the red line were excluded.

B-C. Distributions of per sample total amount (B) and percentage (C) of read pairs (discovery set) or reads (validation set) that are uniquely (and concordantly for the discovery set) mapped to the canFam3 genome. Red line specifies the cutoff (20 million reads for B; 0.3 for C) and samples below the red line were excluded.

D. Distributions of per sample CDS-targeting rate. Red line specifies the cutoff (0.21) and samples below the red line were excluded.

E. Gene expression distributions in each sample. Each line represents a sample, with the TPM value of each of all ~20,000 protein-coding genes plotted.

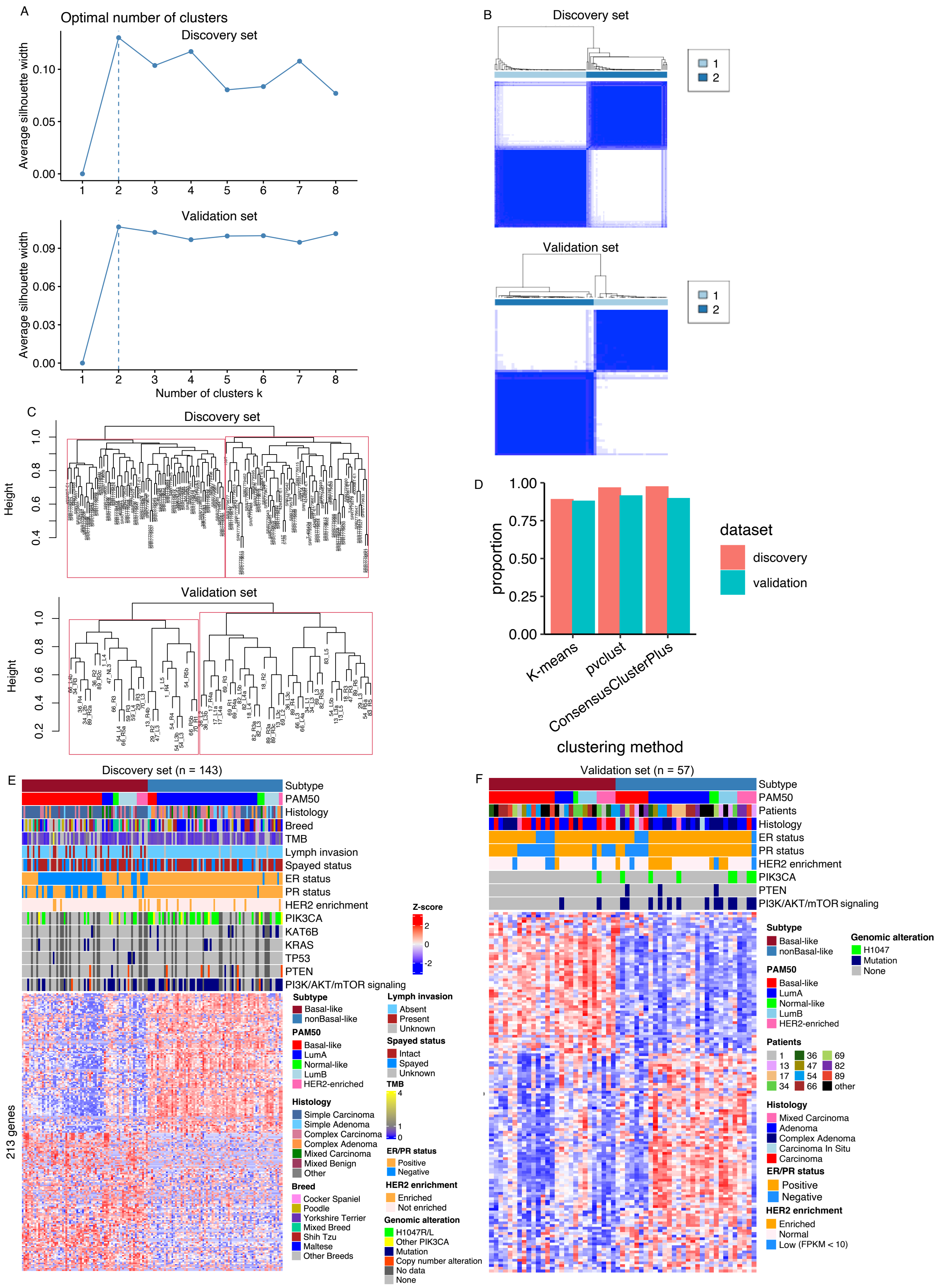
A
Optimal number of clusters
Discovery set
Average silhouette width
0.00
0.05
0.10
Validation set
0.00
0.03
0.06
0.09
1 2 3 4 5 6 7 8
Number of clusters k
B
Discovery set
1
2
Validation set
1
2
C
Discovery set
Height
0.4 0.6 0.8 1.0
Validation set
Height
0.2 0.4 0.6 0.8 1.0
D
proportion
0.00 0.25 0.50 0.75 1.00
K-means
pvclust
ConsensusClusterPlus
clustering method
dataset
discovery
validation
E
Discovery set (n = 143)
Subtype
PAM50
Histology
Breed
TMB
Lymph invasion
Spayed status
ER status
PR status
HER2 enrichment
PIK3CA
KAT6B
KRAS
TP53
PTEN
PI3K/AKT/mTOR signaling
213 genes
Z-score
2
0
-2
Subtype
Basal-like
nonBasal-like
PAM50
Basal-like
LumA
Normal-like
LumB
HER2-enriched
Histology
Simple Carcinoma
Simple Adenoma
Complex Carcinoma
Complex Adenoma
Mixed Carcinoma
Mixed Benign
Other
Breed
Cocker Spaniel
Poodle
Yorkshire Terrier
Mixed Breed
Shih Tzu
Maltese
Other Breeds
Lymph invasion
Absent
Present
Unknown
Spayed status
Intact
Spayed
Unknown
TMB
4
1
0
ER/PR status
Positive
Negative
HER2 enrichment
Enriched
Not enriched
Genomic alteration
H1047R/L
Other PIK3CA
Mutation
Copy number alteration
No data
None
F
Validation set (n = 57)
Subtype
PAM50
Patients
Histology
ER status
PR status
HER2 enrichment
PIK3CA
PTEN
PI3K/AKT/mTOR signaling
Subtype
Basal-like
nonBasal-like
Genomic alteration
H1047
Mutation
None
PAM50
Basal-like
LumA
Normal-like
LumB
HER2-enriched
Patients
1
13
17
34
36
47
54
66
69
82
89
other
Histology
Mixed Carcinoma
Adenoma
Complex Adenoma
Carcinoma In Situ
Carcinoma
ER/PR status
Positive
Negative
HER2 enrichment
Enriched
Normal
Low (FPKM < 10)

Fig. S2. Validation of canine mammary tumor subtyping results shown in Fig. 1 using different strategies and data set; related to Fig. 1 and Table S1.

A-C. K-means (A), consensus clustering (ConsensusClusterPlus) (B), and permutation-based hierarchical clustering (pvclust) (C) were applied using the top 10% most variably expressed genes in samples of the discovery set (top plots) or the validation set (bottom plots). These approaches all yield two subtypes, the same as the NMF strategy shown in Fig. 1.

D. The proportions of samples assigned to the same subtype by each approach indicated in A-C as the NMF strategy shown in Fig. 1.

E-F. Heatmaps of the discovery (E) and validation (F) sets as presented in Fig. 1, with NMF clustering conducted on the top 2,000 most variably expressed genes within each cohort.

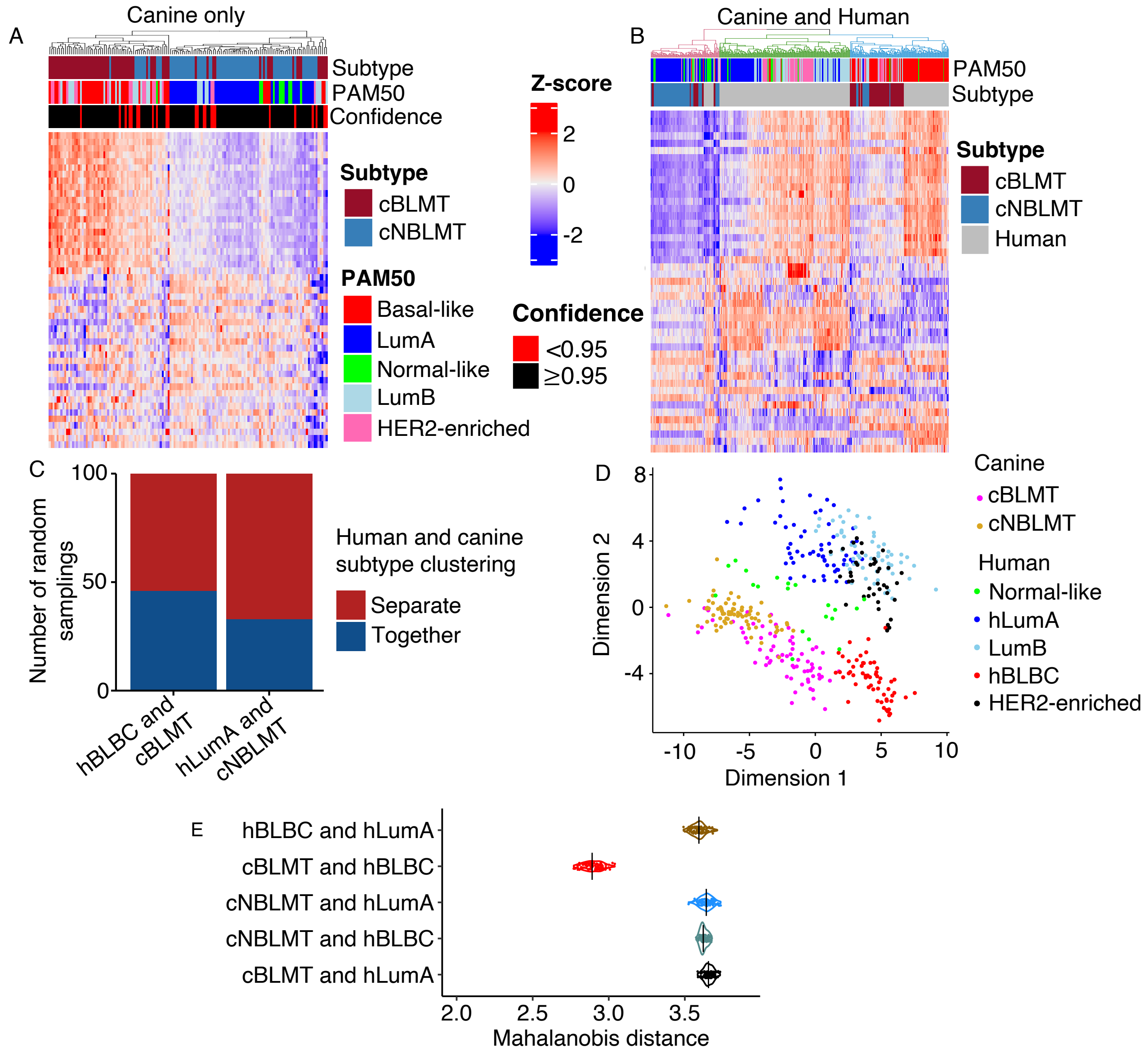
A
Canine only
Subtype
PAM50
Confidence
Subtype
cBLMT
cNBLMT
PAM50
Basal-like
LumA
Normal-like
LumB
HER2-enriched
Z-score
2
0
-2
Confidence
<0.95
≥0.95
B
Canine and Human
PAM50
Subtype
Subtype
cBLMT
cNBLMT
Human
C
Number of random samplings
100
50
0
hBLBC and cBLMT
hLumA and cNBLMT
Human and canine subtype clustering
Separate
Together
D
Dimension 2
Dimension 1
Canine
cBLMT
cNBLMT
Human
Normal-like
hLumA
LumB
hBLBC
HER2-enriched
E
hBLBC and hLumA
cBLMT and hBLBC
cNBLMT and hLumA
cNBLMT and hBLBC
cBLMT and hLumA
2.0
2.5
3.0
3.5
Mahalanobis distance

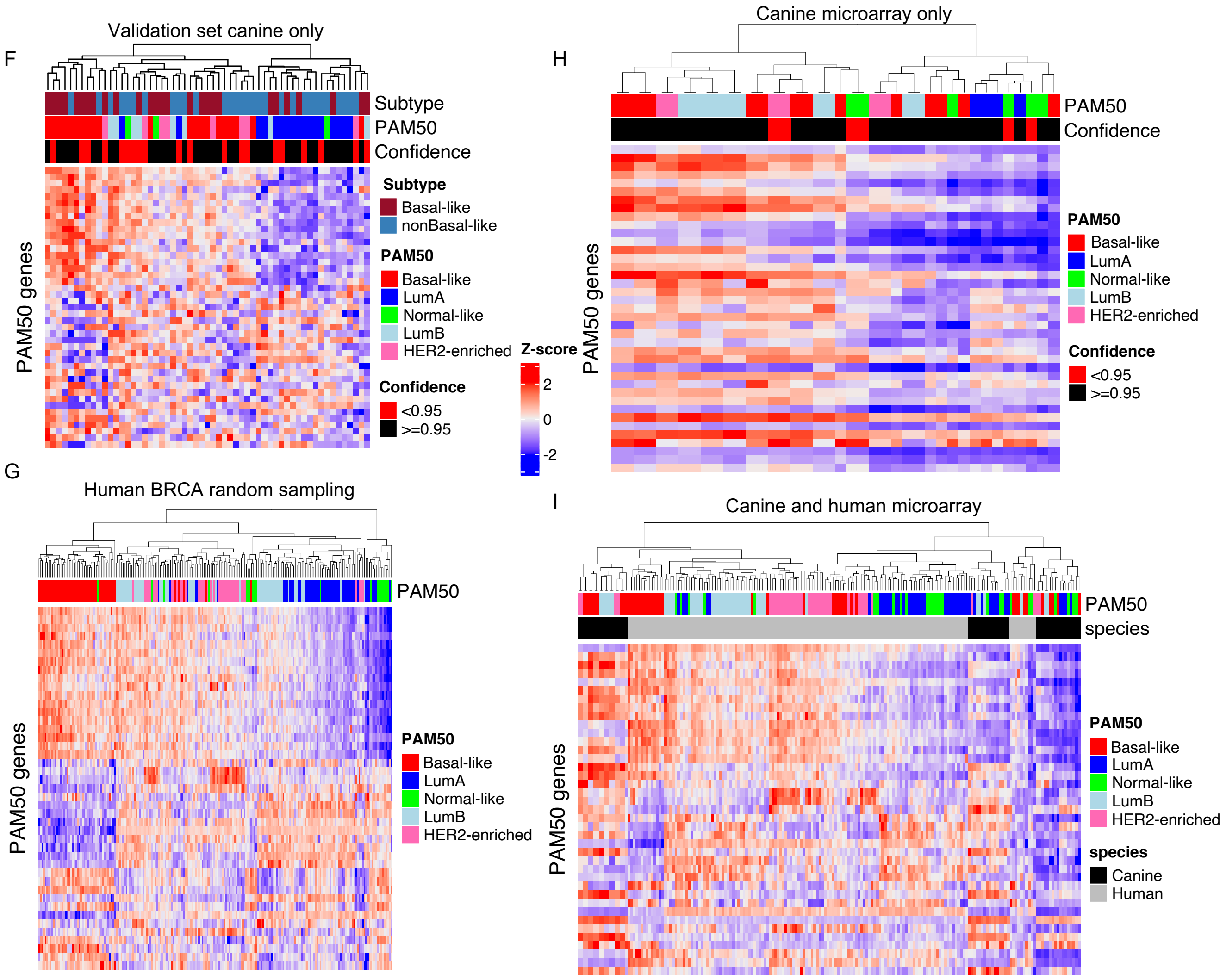
F
Validation set canine only
Subtype
PAM50
Confidence
PAM50 genes
Subtype
Basal-like
nonBasal-like
PAM50
Basal-like
LumA
Normal-like
LumB
HER2-enriched
Confidence
<0.95
>=0.95
Z-score
2
0
-2
H
Canine microarray only
PAM50
Confidence
PAM50 genes
PAM50
Basal-like
LumA
Normal-like
LumB
HER2-enriched
Confidence
<0.95
>=0.95
G
Human BRCA random sampling
PAM50
PAM50 genes
PAM50
Basal-like
LumA
Normal-like
LumB
HER2-enriched
I
Canine and human microarray
PAM50
species
PAM50 genes
PAM50
Basal-like
LumA
Normal-like
LumB
HER2-enriched
species
Canine
Human

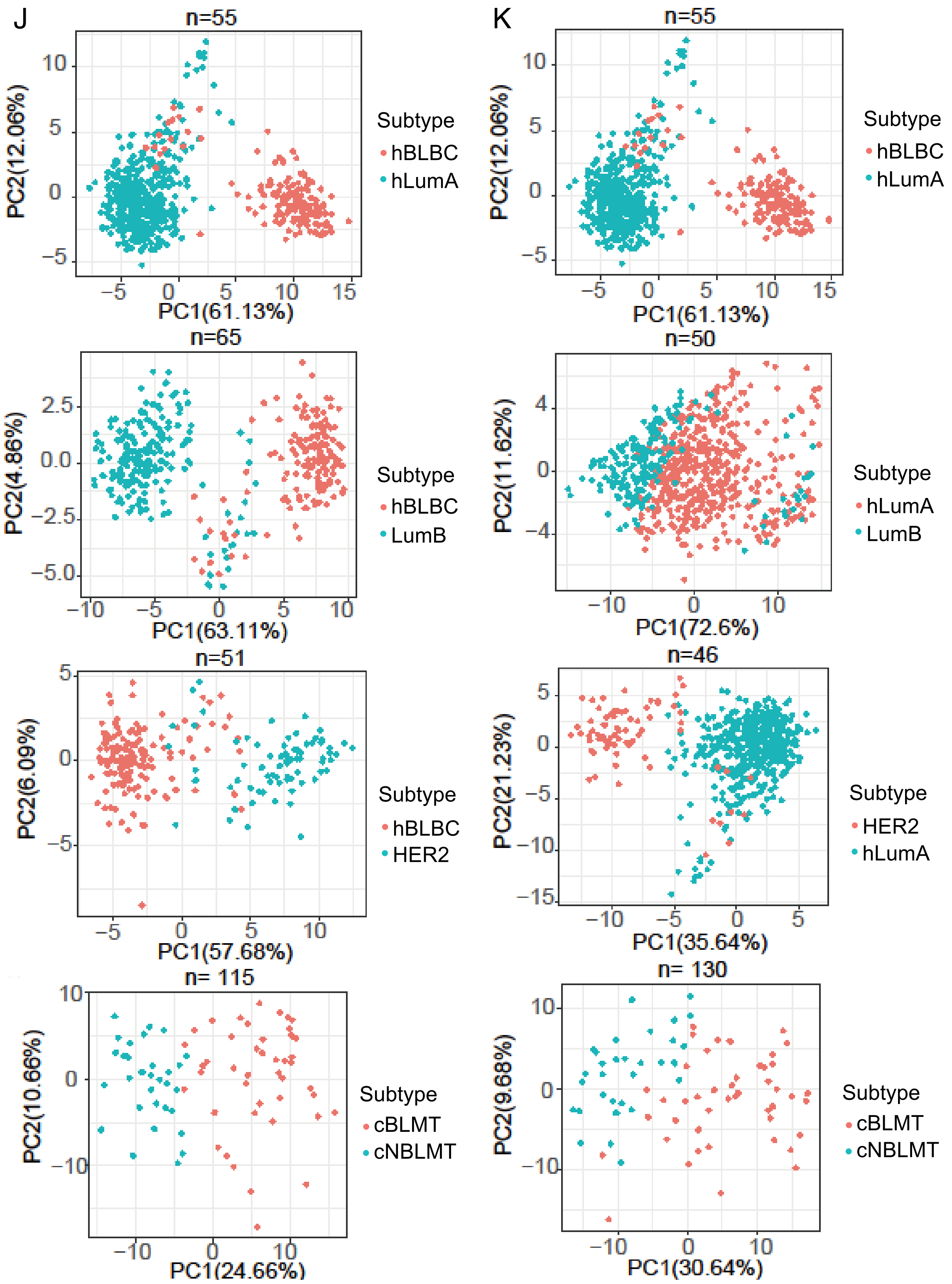
J
K
n=55
PC2(12.06%)
PC1(61.13%)
Subtype
hBLBC
hLumA
n=55
PC2(12.06%)
PC1(61.13%)
Subtype
hBLBC
hLumA
n=65
PC2(4.86%)
PC1(63.11%)
Subtype
hBLBC
LumB
n=50
PC2(11.62%)
PC1(72.6%)
Subtype
hLumA
LumB
n=51
PC2(6.09%)
PC1(57.68%)
Subtype
hBLBC
HER2
n=46
PC2(21.23%)
PC1(35.64%)
Subtype
HER2
hLumA
n= 115
PC2(10.66%)
PC1(24.66%)
Subtype
cBLMT
cNBLMT
n= 130
PC2(9.68%)
PC1(30.64%)
Subtype
cBLMT
cNBLMT

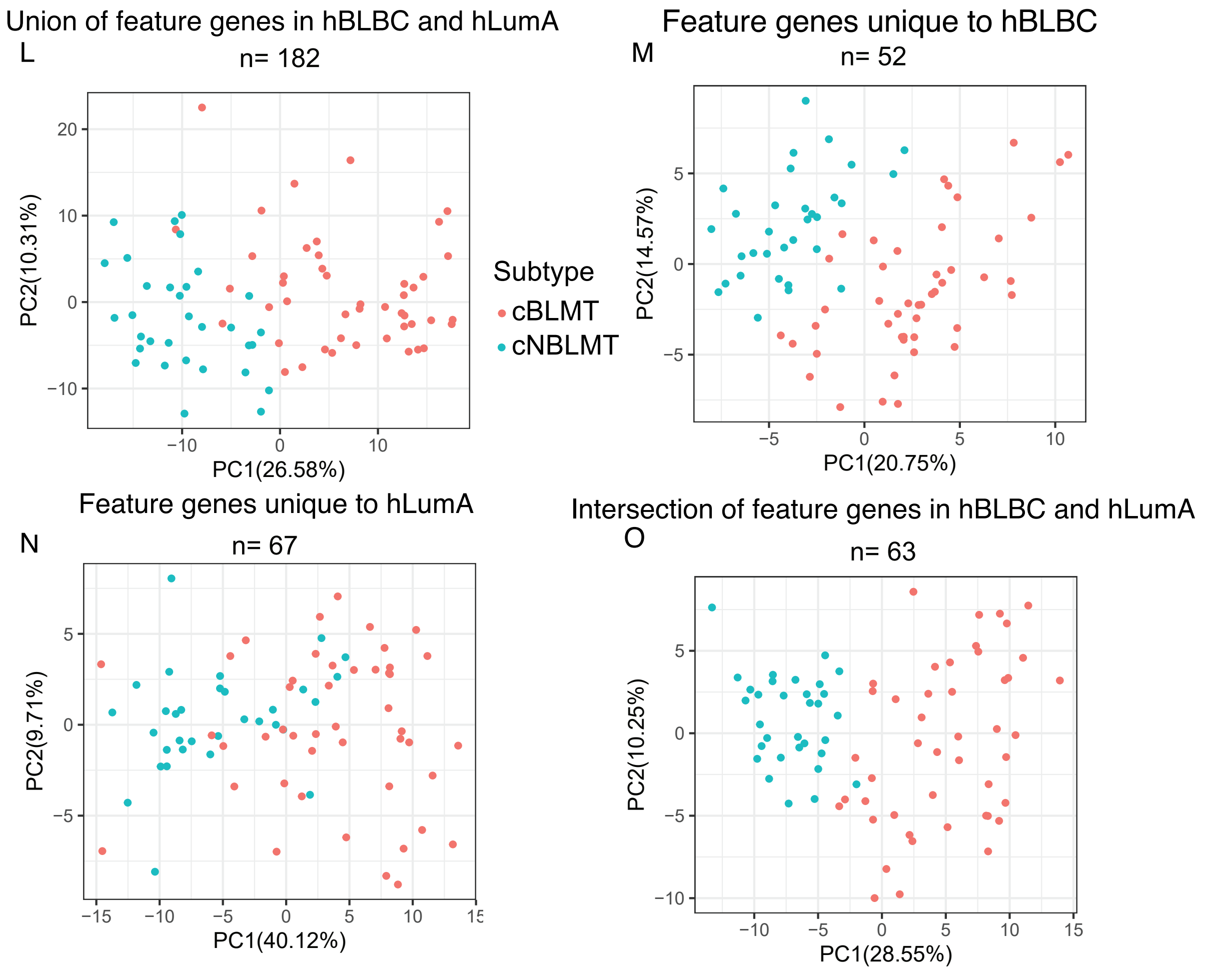

Fig. S3. Dog-alone and cross-species PAM50 classification using canFam4 and its annotation; hBLBC- and hLumA feature gene identification via machine learning; related to Fig. 2 and Table S2.

A. PAM50 classification of 143 subtyped canine mammary tumors using 49 PAM50 genes from canFam4, as presented in Fig. 2A.

B. An example of cross-species PAM50 classification, as presented in Fig. 2B, with 49 PAM50 genes from canFam4.

C. Bar plot showing the number of random samplings in which hBLBC and cBLMT or hLumA and cNBLMT clustered together or separately, as presented in Fig. 2C, using the 49 PAM50 genes from canFam4.

D. Multidimensional scaling plot of the cross-species PAM50 classification shown in B, plotted as in Fig. 2D, using the 49 PAM50 genes from canFam4.

E. Violin plot indicating the distribution of the mahalanobis distances between the centers of two subtypes on the multidimensional scaled plot, as presented in Fig. 2E, using the 49 PAM50 genes from canFam4.

F. PAM50 classification of the 57 tumors of the validation set, presented as described in Fig. 2A.

G. PAM50 classification of 267 human breast tumors sampled from TCGA as described in the Methods section. The 50 PAM50 genes were used, and the tumor subtypes shown were from cBioportal.

H. PAM50 classification of 40 canine tumors from two gene expression microarray studies [1, 2]. Log_2-transformed expression values of 40 genes out of the 50 PAM50 genes were used (see Methods).

I. An example of cross-species PAM50 classification using canine and human microarray data. Human tumors (30 tumors per PAM50 subtype) were randomly sampled from a microarray data study [3] as described in the Methods section. The figure is presented as described for Fig. 2B.

J. Principal component analysis (PCA) plots using machine learning (ML)-selected feature gene sets that separate hBLBC from hLumA, LumB, and HER2 (top three plots, from top to bottom, respectively) breast cancer samples from TCGA. Bottom PCA plot is done using all 143 subtyped canine mammary tumors with the union of feature genes indicated in the top three plots (115 genes in total), with the number of feature genes specified by “n”.

K. PCA plots using ML-selected feature gene sets that separate hLumA from hBLBC, LumB, and HER2 (top three plots, from top to bottom, respectively) breast cancer samples from TCGA. Bottom PCA plot is done using all 143 subtyped canine mammary tumors with the union of feature genes indicated in the top three plots (n = 130).

L-O. PCA plots of all 143 subtyped canine mammary tumors using different combinations of ML-selected feature gene sets, including hBLBC and hLumA feature genes combined (L), hBLBC-unique feature genes (M), hLumA-unique feature genes (N), and feature genes shared by both hBLBC and hLumA (O).

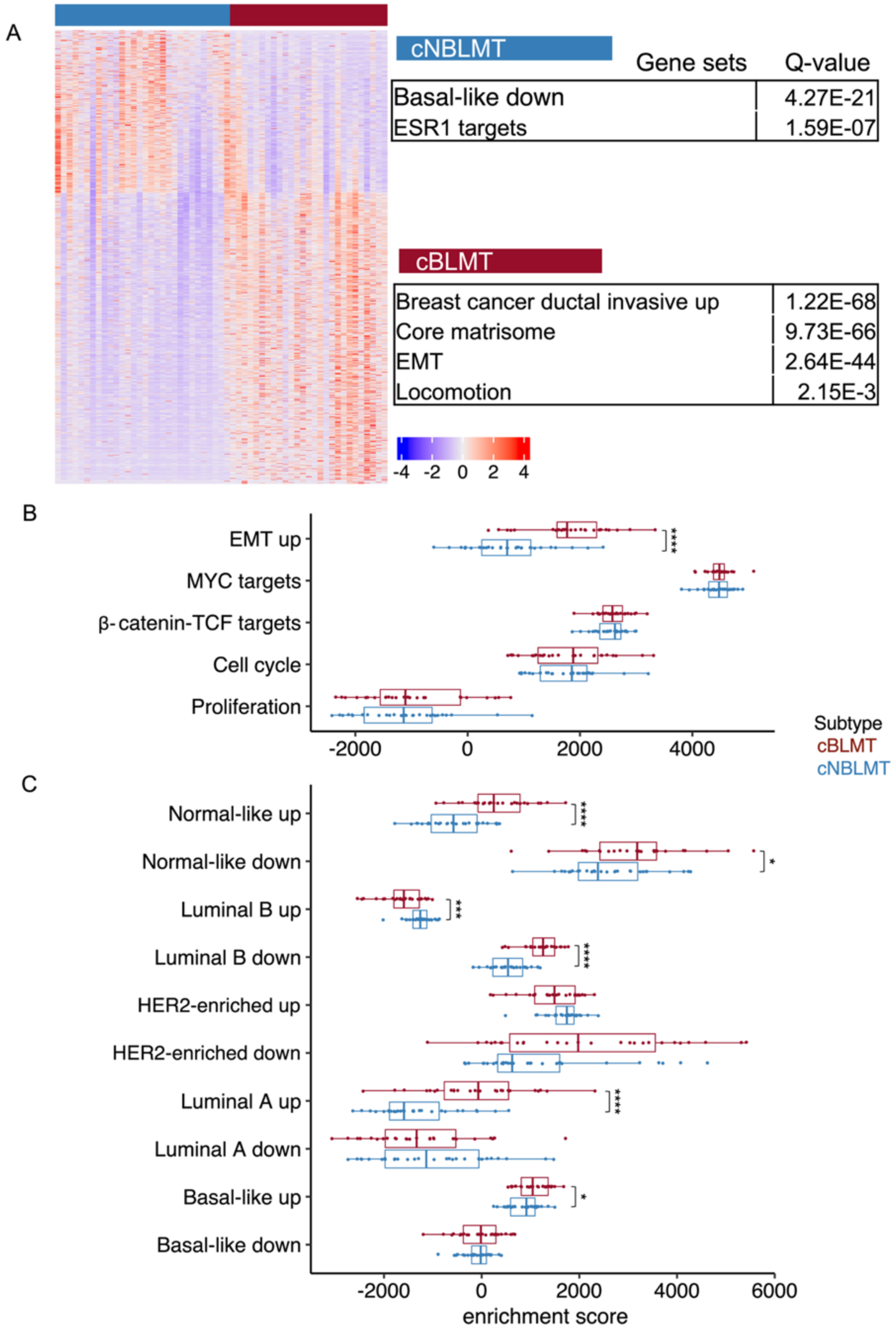
A
cNBLMT
Gene sets
Q-value
Basal-like down 4.27E-21
ESR1 targets 1.59E-07
cBLMT
Breast cancer ductal invasive up 1.22E-68
Core matrisome 9.73E-66
EMT 2.64E-44
Locomotion 2.15E-3
-4 -2 0 2 4
B
EMT up
MYC targets
β-catenin-TCF targets
Cell cycle
Proliferation
-2000 0 2000 4000
Subtype
cBLMT
cNBLMT
C
Normal-like up
Normal-like down
Luminal B up
Luminal B down
HER2-enriched up
HER2-enriched down
Luminal A up
Luminal A down
Basal-like up
Basal-like down
-2000 0 2000 4000 6000
enrichment score

Fig. S4. Differentially expressed (DE) gene analysis indicates enrichment of hBLBC signatures in cBLMT of the validation set; related to Fig. 3 and Table S3.

A. Heatmap of the row scaled log_2(TPM) values of the 761 DE genes between cBLMT and cNBLMT of the validation set, presented as described for Fig. 3A.

B & C. Distributions of ssGSEA scores, presented as described for Figs. 3B-C.

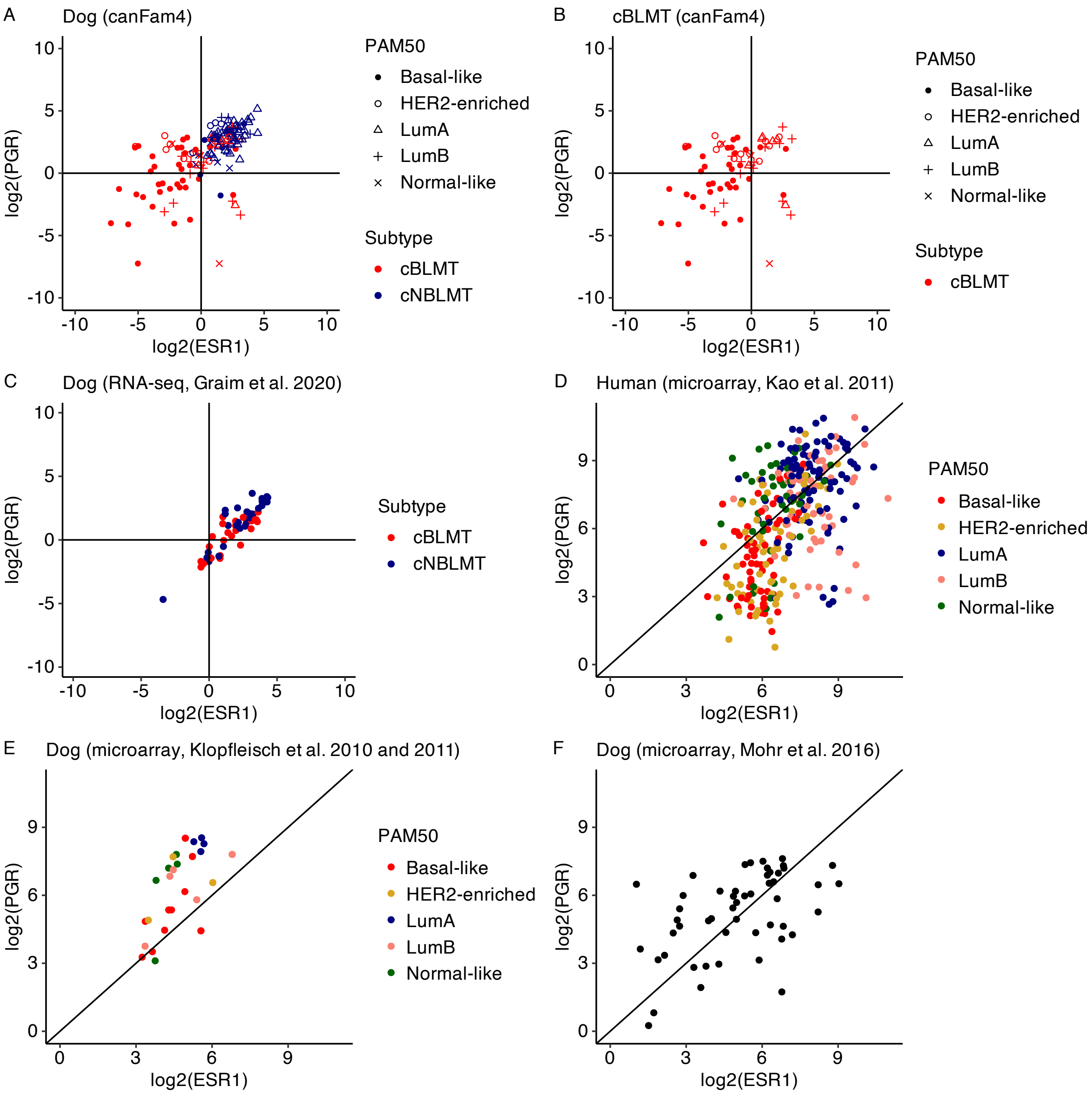

Fig. S5. Canine tumors, especially cBLMTs, express *PGR* more abundantly than hBLBCs; related to Fig. 4 and Table S4.

A-B. Scatter plots showing the $\log_2$(FPKM) values of *ESR1* and *PGR* for both cBLMTs and cNBLMTs (n=143) (A), and only cBLMTs (n=69) with the PAM50 subtype (B), using *ESR1* and *PGR* values from canFam4.

C. Scatter plot showing the log_2(FPKM) values of *ESR1* and *PGR* for canine tumors of the validation set (n=57) [4].

D-F. Scatter plots showing the log_2-tranformed expression values of *ESR1* and *PGR* from microarray studies of human breast cancers (n=327) [3] (D), as well as of canine mammary tumors by one group (n=40) (E) [1, 2] and by another group (n=51) (F) [5].

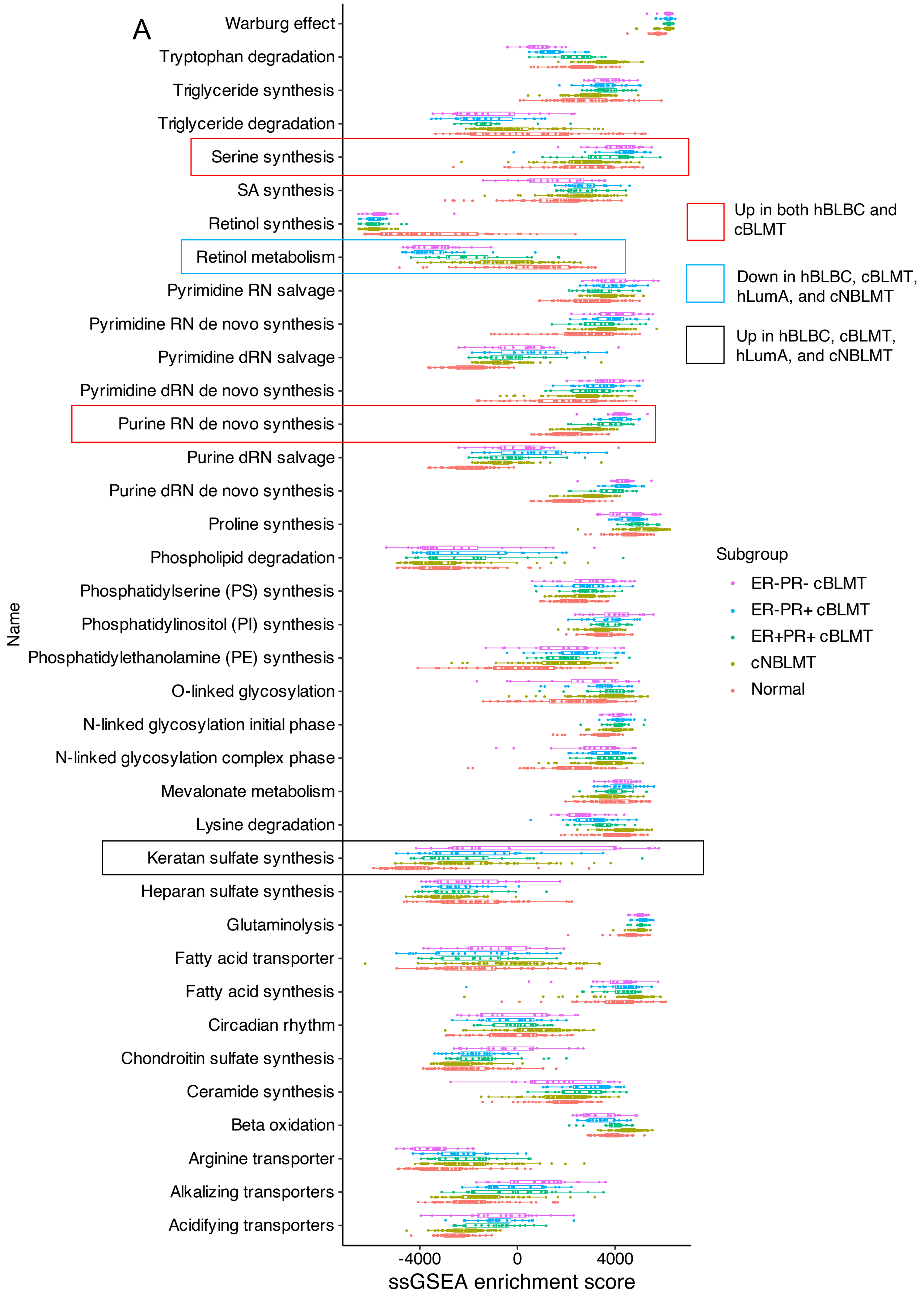

A
Warburg effect
Tryptophan degradation
Triglyceride synthesis
Triglyceride degradation
Serine synthesis
SA synthesis
Retinol synthesis
Retinol metabolism
Pyrimidine RN salvage
Pyrimidine RN de novo synthesis
Pyrimidine dRN salvage
Pyrimidine dRN de novo synthesis
Purine RN de novo synthesis
Purine dRN salvage
Purine dRN de novo synthesis
Proline synthesis
Phospholipid degradation
Phosphatidylserine (PS) synthesis
Phosphatidylinositol (PI) synthesis
Phosphatidylethanolamine (PE) synthesis
O-linked glycosylation
N-linked glycosylation initial phase
N-linked glycosylation complex phase
Mevalonate metabolism
Lysine degradation
Keratan sulfate synthesis
Heparan sulfate synthesis
Glutaminolysis
Fatty acid transporter
Fatty acid synthesis
Circadian rhythm
Chondroitin sulfate synthesis
Ceramide synthesis
Beta oxidation
Arginine transporter
Alkalizing transporters
Acidifying transporters
Name
-4000
0
4000
ssGSEA enrichment score
Up in both hBLBC and cBLMT
Down in hBLBC, cBLMT, hLumA, and cNBLMT
Up in hBLBC, cBLMT, hLumA, and cNBLMT
Subgroup
ER-PR- cBLMT
ER-PR+ cBLMT
ER+PR+ cBLMT
cNBLMT
Normal

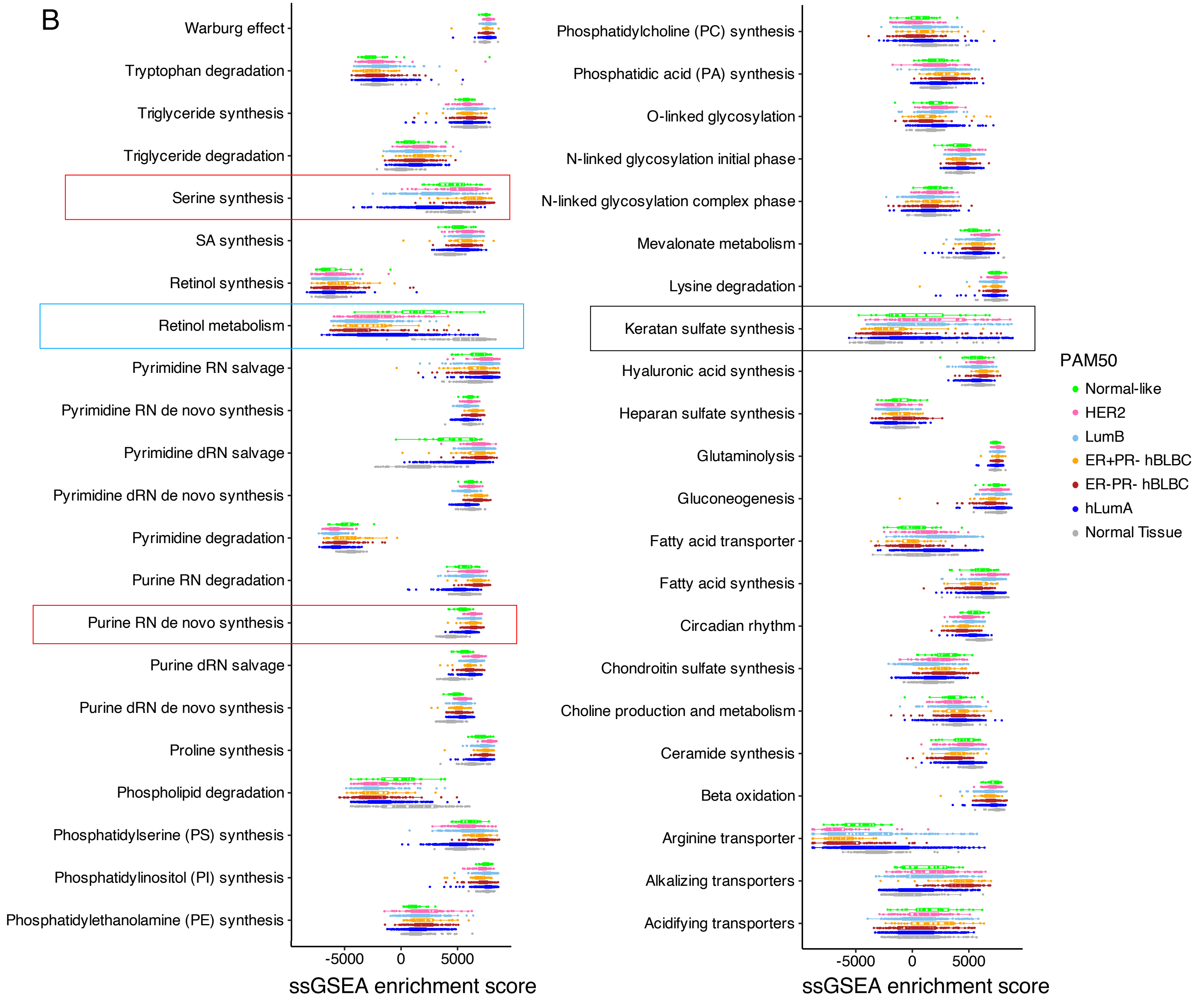

B
Warburg effect
Tryptophan degradation
Triglyceride synthesis
Triglyceride degradation
Serine synthesis
SA synthesis
Retinol synthesis
Retinol metabolism
Pyrimidine RN salvage
Pyrimidine RN de novo synthesis
Pyrimidine dRN salvage
Pyrimidine dRN de novo synthesis
Pyrimidine degradation
Purine RN degradation
Purine RN de novo synthesis
Purine dRN salvage
Purine dRN de novo synthesis
Proline synthesis
Phospholipid degradation
Phosphatidylserine (PS) synthesis
Phosphatidylinositol (PI) synthesis
Phosphatidylethanolamine (PE) synthesis
-5000
0
5000
ssGSEA enrichment score
Phosphatidylcholine (PC) synthesis
Phosphatidic acid (PA) synthesis
O-linked glycosylation
N-linked glycosylation initial phase
N-linked glycosylation complex phase
Mevalonate metabolism
Lysine degradation
Keratan sulfate synthesis
Hyaluronic acid synthesis
Heparan sulfate synthesis
Glutaminolysis
Gluconeogenesis
Fatty acid transporter
Fatty acid synthesis
Circadian rhythm
Chondroitin sulfate synthesis
Choline production and metabolism
Ceramide synthesis
Beta oxidation
Arginine transporter
Alkalizing transporters
Acidifying transporters
-5000
0
5000
ssGSEA enrichment score
PAM50
Normal-like
HER2
LumB
ER+PR- hBLBC
ER-PR- hBLBC
hLumA
Normal Tissue

Fig. S6. Purine de novo synthesis and serine synthesis are more activated in cBLMTs and hBLBCs, compared to normal mammary tissues; related to Fig. 5 and Table S5.

A-B. Distributions of ssGSEA enrichment scores for metabolic pathways using signature genes identified for each pathway as described [6], in canine tumor subgroups and subtypes (A) and human PAM50 subtypes (B). Each dot represents a sample. Significantly up- or downregulated pathways are identified with BH adjusted $p < 0.05$ and $|\log_2(fold\ change)| > 0.5$, by comparing the cancer types with normal samples.

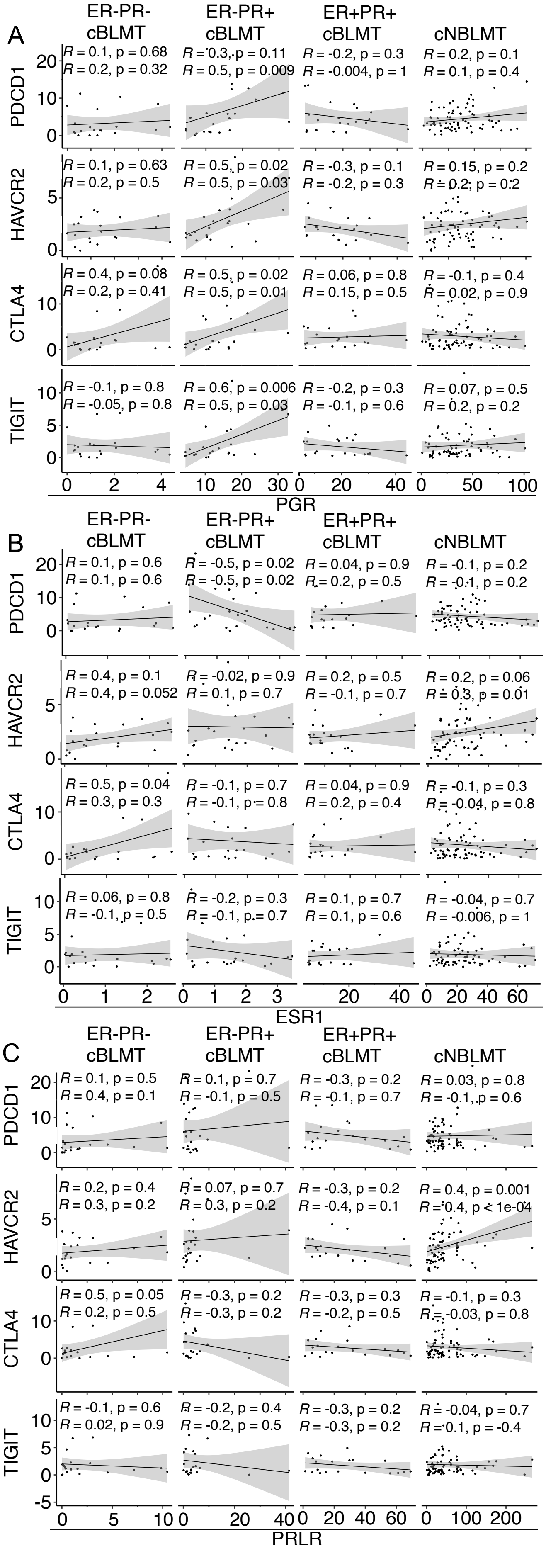
A
ER-PR- cBLMT
ER-PR+ cBLMT
ER+PR+ cBLMT
cNBLMT
PDCD1
R = 0.1, p = 0.68
R = 0.2, p = 0.32
R = 0.3, p = 0.11
R = 0.5, p = 0.009
R = -0.2, p = 0.3
R = -0.004, p = 1
R = 0.2, p = 0.1
R = 0.1, p = 0.4
HAVCR2
R = 0.1, p = 0.63
R = 0.2, p = 0.5
R = 0.5, p = 0.02
R = 0.5, p = 0.03
R = -0.3, p = 0.1
R = -0.2, p = 0.3
R = 0.15, p = 0.2
R = 0.2, p = 0.2
CTLA4
R = 0.4, p = 0.08
R = 0.2, p = 0.41
R = 0.5, p = 0.02
R = 0.5, p = 0.01
R = 0.06, p = 0.8
R = 0.15, p = 0.5
R = -0.1, p = 0.4
R = 0.02, p = 0.9
TIGIT
R = -0.1, p = 0.8
R = -0.05, p = 0.8
R = 0.6, p = 0.006
R = 0.5, p = 0.03
R = -0.2, p = 0.3
R = -0.1, p = 0.6
R = 0.07, p = 0.5
R = 0.2, p = 0.2
PGR
B
ER-PR- cBLMT
ER-PR+ cBLMT
ER+PR+ cBLMT
cNBLMT
PDCD1
R = 0.1, p = 0.6
R = 0.1, p = 0.6
R = -0.5, p = 0.02
R = -0.5, p = 0.02
R = 0.04, p = 0.9
R = 0.2, p = 0.5
R = -0.1, p = 0.2
R = -0.1, p = 0.2
HAVCR2
R = 0.4, p = 0.1
R = 0.4, p = 0.052
R = -0.02, p = 0.9
R = 0.1, p = 0.7
R = 0.2, p = 0.5
R = -0.1, p = 0.7
R = 0.2, p = 0.06
R = 0.3, p = 0.01
CTLA4
R = 0.5, p = 0.04
R = 0.3, p = 0.3
R = -0.1, p = 0.7
R = -0.1, p = 0.8
R = 0.04, p = 0.9
R = 0.2, p = 0.4
R = -0.1, p = 0.3
R = -0.04, p = 0.8
TIGIT
R = 0.06, p = 0.8
R = -0.1, p = 0.5
R = -0.2, p = 0.3
R = -0.1, p = 0.7
R = 0.1, p = 0.7
R = 0.1, p = 0.6
R = -0.04, p = 0.7
R = -0.006, p = 1
ESR1
C
ER-PR- cBLMT
ER-PR+ cBLMT
ER+PR+ cBLMT
cNBLMT
PDCD1
R = 0.1, p = 0.5
R = 0.4, p = 0.1
R = 0.1, p = 0.7
R = -0.1, p = 0.5
R = -0.3, p = 0.2
R = -0.1, p = 0.7
R = 0.03, p = 0.8
R = -0.1, p = 0.6
HAVCR2
R = 0.2, p = 0.4
R = 0.3, p = 0.2
R = 0.07, p = 0.7
R = 0.3, p = 0.2
R = -0.3, p = 0.2
R = -0.4, p = 0.1
R = 0.4, p = 0.001
R = 0.4, p < 1e-04
CTLA4
R = 0.5, p = 0.05
R = 0.2, p = 0.5
R = -0.3, p = 0.2
R = -0.3, p = 0.2
R = -0.3, p = 0.3
R = -0.2, p = 0.5
R = -0.1, p = 0.3
R = -0.03, p = 0.8
TIGIT
R = -0.1, p = 0.6
R = 0.02, p = 0.9
R = -0.2, p = 0.4
R = -0.2, p = 0.5
R = -0.3, p = 0.2
R = -0.3, p = 0.2
R = -0.04, p = 0.7
R = 0.1, p = -0.4
PRLR

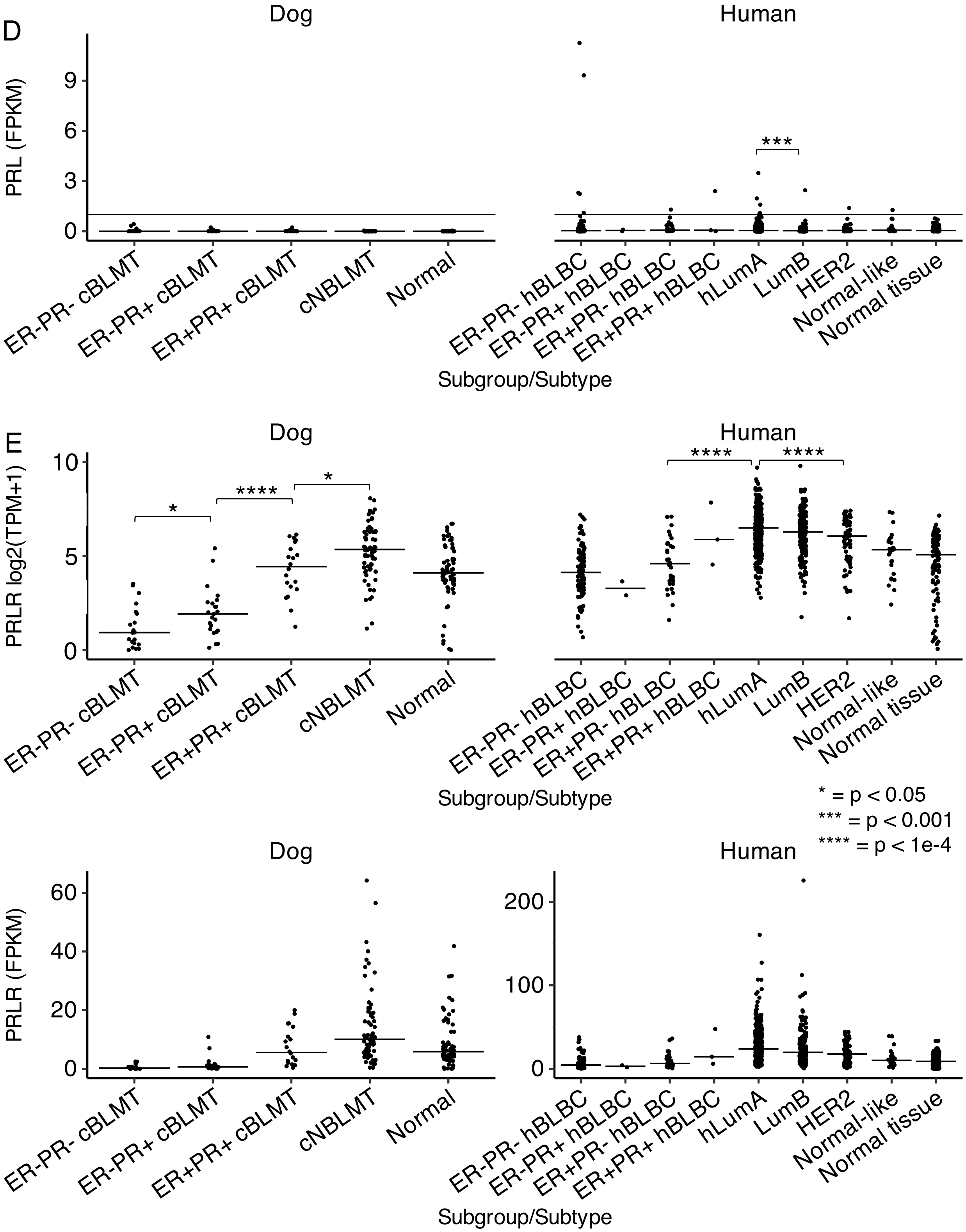
D
E
Dog
Human
PRL (FPKM)
PRLR log2(TPM+1)
PRLR (FPKM)
ER-PR- cBLMT
ER-PR+ cBLMT
ER+PR+ cBLMT
cNBLMT
Normal
ER-PR- hBLBC
ER-PR+ hBLBC
ER+PR- hBLBC
ER+PR+ hBLBC
hLumA
LumB
HER2
Normal-like
Normal tissue
Subgroup/Subtype
* = p < 0.05
*** = p < 0.001
**** = p < 1e-4

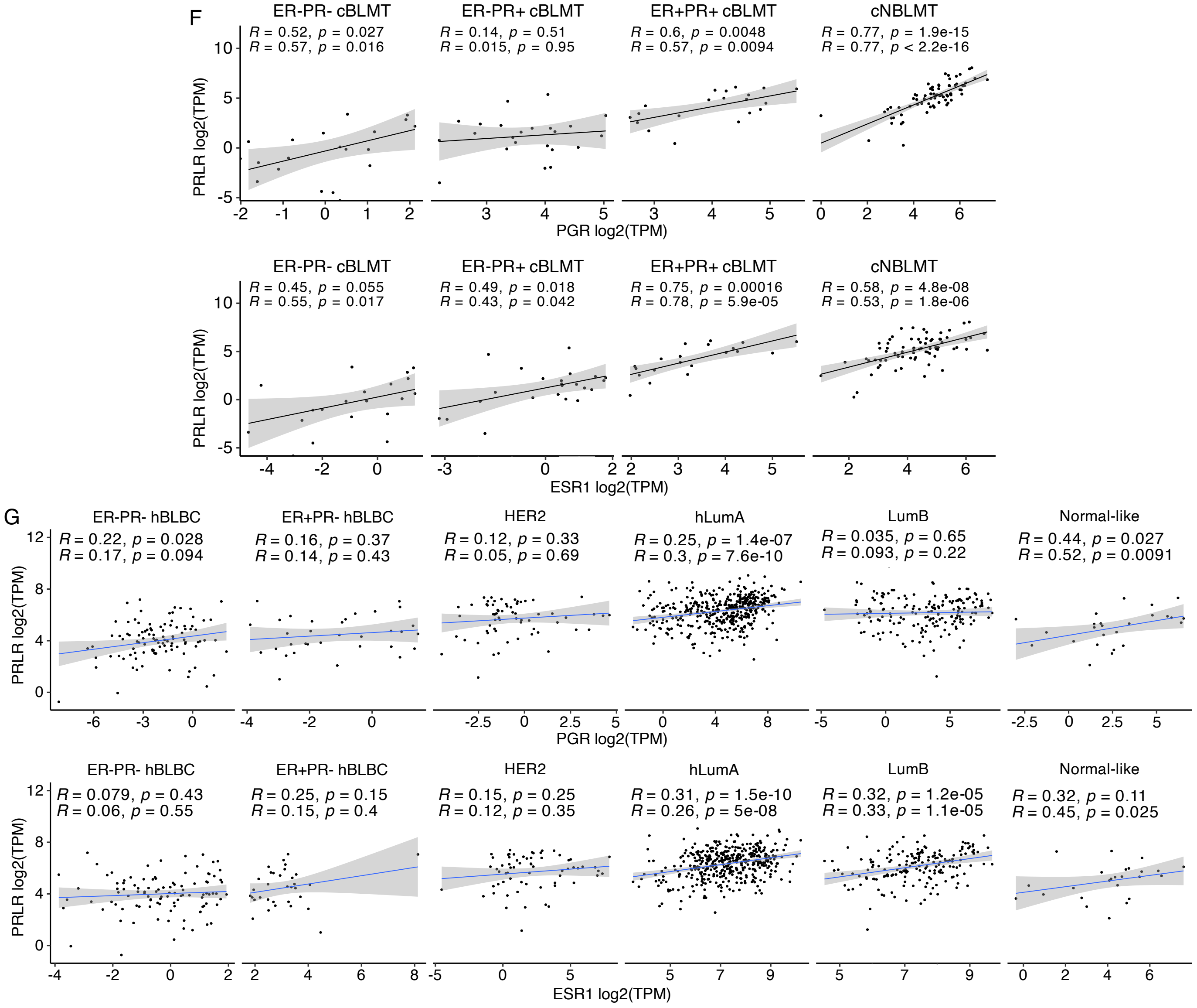

Fig. S7. ***PGR,* but not *ESR1* or *PRLR,* correlates with several T-cell exhaustion signature genes in mRNA expression in ER-PR+ cBLMTs; *PRL* and *PRLR* expression in canine and human tumors;** related to Fig. 6.

A-C. Pearson (top) and Spearman (bottom) correlation analysis between *PGR* (A), *ESR1* (B), or *PRLR* (C) and *PDCD1*, *HAVCR2, CTLA4*, or *TIGIT* in mRNA expression in each canine subgroup and subtype shown. Correlation coefficients, p-values, and linear regression lines are indicated.

D. Distribution of FPKM expression values of *PRL* in canine and human subgroups and subtypes. P-values are derived from Wilcox tests. The black line specifies $FPKM = 1$, the threshold for classifying a gene as being expressed, which no canine and few human samples pass. Human samples are from TCGA breast cancer study.

E. Distributions of $\log_2$(TPM+1) (top) and FPKM (bottom) expression values of *PRLR* in canine and human subgroups and subtypes. P-values are derived from Wilcox tests. Human samples are from TCGA breast cancer study.

F. Pearson (top) and Spearman (bottom) correlation analysis between *PGR* (top row) or *ESR1* (bottom row) and *PRLR* in mRNA expression, in each canine subgroup and subtype shown. Correlation coefficients, p-values, and linear regression lines are indicated.

G. Pearson (top) and Spearman (bottom) correlation analysis between *PGR* (top) or *ESR1* (bottom) and *PRLR* in mRNA expression, in each human subtype shown. Correlation coefficients, p-values, and linear regression lines are indicated. Human samples are from TCGA breast cancer study.

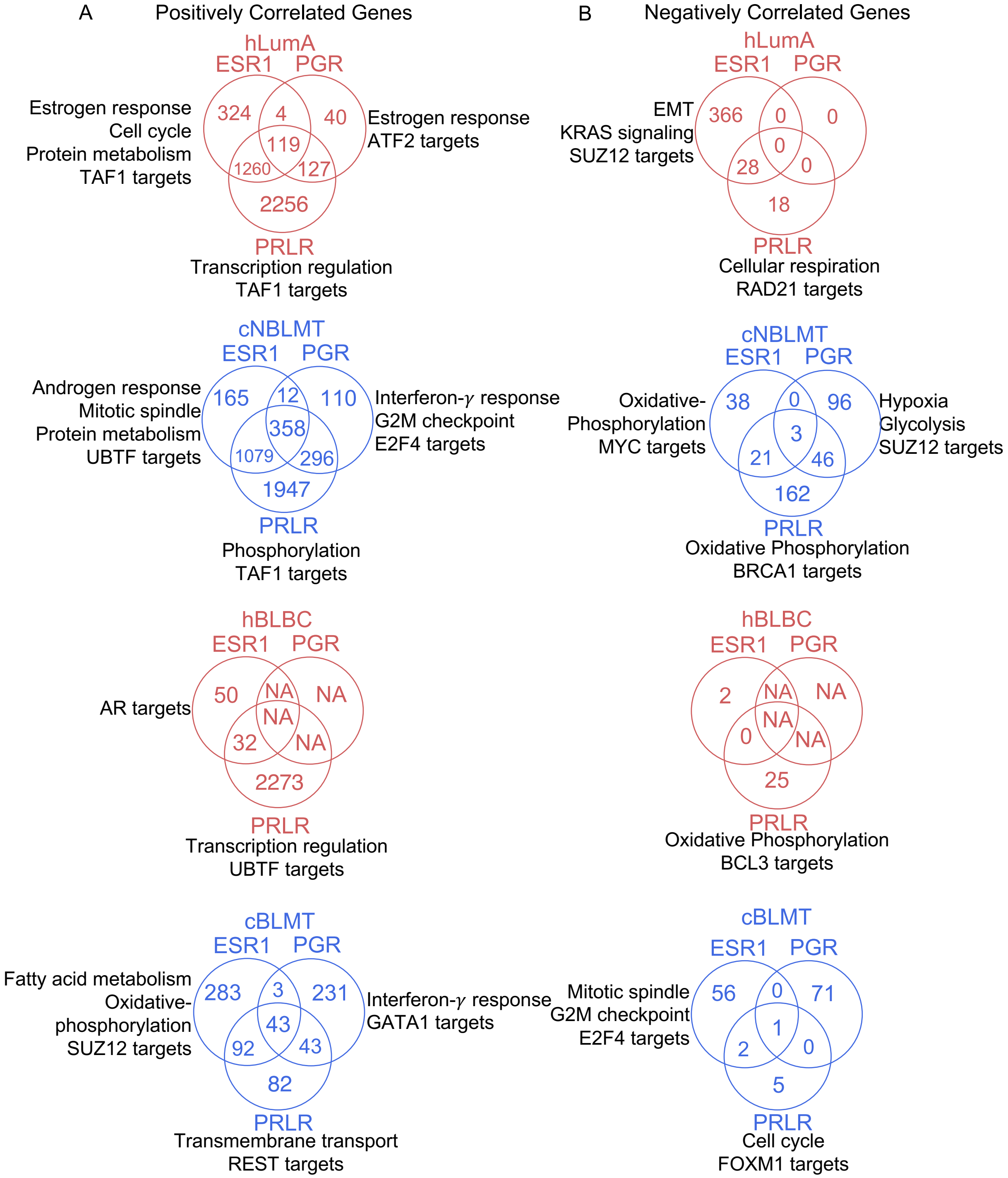

Fig. S8. *PRLR* positively correlates with many genes in mRNA expression, but is not associated with gene silencing in cBLMT; related to Fig. 7 and Table S6.

A-B. Venn diagrams of genes positively (A) or negatively (B) correlated with *PGR, ESR1*, and/or *PRLR*, using the same thresholds as described for Fig. 7 (both Pearson

and Spearman correlation coefficients $R > 0.3$ for positively correlated genes or $R < -0.3$ for negatively correlated genes, and both Pearson and Spearman BH-adjusted $p < 0.05$). Indicated are also the top enriched functions of genes that are correlated with only *ESR1*, *PGR,* or *PRLR.*

1. Klopfleisch R, Lenze D, Hummel M, Gruber AD: **The metastatic cascade is reflected in the transcriptome of metastatic canine mammary carcinomas**. *Vet J* 2011, **190**(2):236-243.
2. Klopfleisch R, Lenze D, Hummel M, Gruber AD: **Metastatic canine mammary carcinomas can be identified by a gene expression profile that partly overlaps with human breast cancer profiles**. *BMC Cancer* 2010, **10**:618.
3. Kao KJ, Chang KM, Hsu HC, Huang AT: **Correlation of microarray-based breast cancer molecular subtypes and clinical outcomes: implications for treatment optimization**. *BMC Cancer* 2011, **11**:143.
4. Graim K, Gorenshteyn D, Robinson DG, Carriero NJ, Cahill JA, Chakrabarti R, Goldschmidt MH, Durham AC, Funk J, Storey JD *et al*: **Modeling molecular development of breast cancer in canine mammary tumors**. *Genome Res* 2020.
5. Mohr A, Luder Ripoli F, Hammer SC, Willenbrock S, Hewicker-Trautwein M, Kielbowicz Z, Murua Escobar H, Nolte I: **Hormone Receptor Expression Analyses in Neoplastic and Non-Neoplastic Canine Mammary Tissue by a Bead Based Multiplex Branched DNA Assay: A Gene Expression Study in Fresh Frozen and Formalin-Fixed, Paraffin-Embedded Samples**. *PLoS One* 2016, **11**(9):e0163311.
6. Sun H, Zhou Y, Skaro MF, Wu Y, Qu Z, Mao F, Zhao S, Xu Y: **Metabolic Reprogramming in Cancer Is Induced to Increase Proton Production**. *Cancer Res* 2020, **80**(5):1143-1155.
